# Impact of Resuscitation Status and Cardiac Arrest Location on Survival and Neurological Outcomes in Acute Coronary Syndrome Patients Undergoing Coronary Angiography

**DOI:** 10.3390/jcm15145645

**Published:** 2026-07-18

**Authors:** Artiomas Širvys, Mindaugas Smetaninas, Vilhelmas Bajoras, Arvydas Baranauskas

**Affiliations:** 1 Center of Cardiology and Angiology, Vilnius University Hospital Santaros Klinikos, LT-08661 Vilnius, Lithuania; artiomas.sirvys@santa.lt (A.Š.); vilhelmas.bajoras@santa.lt (V.B.); 2 Faculty of Medicine, Vilnius University, LT-03101 Vilnius, Lithuania; mindaugas.smetaninas@mf.stud.vu.lt

**Keywords:** cardiac arrest, acute coronary syndrome, resuscitation

## Abstract

**Background:** Cardiac arrest complicating acute coronary syndrome (ACS) is associated with high mortality and neurological morbidity despite advances in percutaneous coronary intervention (PCI) and post-resuscitation care. This study evaluated clinical outcomes in ACS patients presenting with cardiac arrest undergoing invasive coronary angiography, with particular focus on resuscitation status before catheterization and location of cardiac arrest. **Methods:** A retrospective analysis of 15,595 ACS patients undergoing coronary angiography between 2014 and 2025 identified two cohorts. The first cohort included 131 patients stratified according to mechanical cardiac activity upon arrival to the catheterization laboratory: previously resuscitated patients (RES, *n* = 109) and patients in refractory cardiac arrest requiring automatic resuscitation devices (ARD, *n* = 22). The second cohort included 159 patients grouped by arrest location: out-of-hospital (OHCA, *n* = 83), in-hospital (IHCA, *n* = 48), and catheterization laboratory cardiac arrest (CLCA, *n* = 28). The primary outcomes were in-hospital mortality and neurological status after resuscitation. **Results:** In-hospital mortality was significantly higher in the ARD group compared with the RES group (86.4% vs. 39.4%, *p* < 0.001). Survivors in the RES group more frequently achieved favorable neurological recovery (66.2%). Mean resuscitation duration and admission lactate levels were significantly greater in the ARD group (66.1 vs. 21.9 min, *p* < 0.001; 10.7 vs. 7.7 mmol/L, *p* = 0.006). According to arrest location, mortality was highest in the CLCA group (78.6%), followed by IHCA (60.4%) and OHCA (39.8%) (*p* < 0.001). Despite high mortality, all surviving CLCA patients had favorable neurological outcomes. Culprit coronary vessel distribution was not associated with mortality or neurological outcome. **Conclusions:** In ACS patients with cardiac arrest, ongoing refractory arrest during coronary angiography and cardiac arrest occurring in the catheterization laboratory were associated with markedly increased mortality. Successful resuscitation prior to catheterization was associated with significantly better survival and neurological recovery.

## 1. Introduction

Cardiac arrest complicating acute coronary syndrome (ACS) represents one of the most severe clinical presentations in interventional cardiology and is associated with high mortality and substantial neurological morbidity. Out-of-hospital cardiac arrest occurs in approximately 67–170 per 100,000 inhabitants annually in Europe, and survival remains low despite advances in emergency medical systems and post-resuscitation care [1].

Coronary artery occlusion is a frequent underlying cause of cardiac arrest, and urgent coronary angiography with percutaneous coronary intervention (PCI) has become a cornerstone of management when ACS is suspected. However, patients presenting with cardiac arrest remain a particularly high-risk population. Studies have demonstrated significantly higher short-term mortality among ACS patients with cardiac arrest compared with those without arrest, even in the era of contemporary PCI [2]. In large observational analyses, mortality among patients undergoing PCI after out-of-hospital cardiac arrest may approach 30%, with neurological injury representing a major determinant of outcome [3].

Despite advances in reperfusion therapy and critical care, the optimal management strategy for patients presenting with cardiac arrest and suspected ACS remains debated, particularly in complex or frail patients. Clinical outcomes appear to depend not only on successful revascularization but also on factors such as the location of cardiac arrest, duration of resuscitation, and the patient’s physiological status upon arrival to the catheterization laboratory.

Therefore, the aim of this study was to evaluate clinical outcomes in patients with ACS complicated by cardiac arrest undergoing invasive coronary angiography, with particular attention to resuscitation status at arrival to the catheterization laboratory and the location where cardiac arrest occurred, and their association with in-hospital mortality and neurological outcomes.

## 2. Materials and Methods

A total of 15,595 patients with acute coronary syndrome (ACS) who underwent invasive coronary angiography between 2014 and 2025 were retrospectively screened.

Two cohorts were extracted. The first cohort included 131 patients, who were categorized according to the presence of mechanical cardiac activity in the catheterization laboratory. The first group consisted of patients who were resuscitated prior to the procedure and were classified as already resuscitated (RES). The second group consisted of patients in refractory cardiac arrest requiring automatic resuscitation devices (ARD). These patients had no return of spontaneous mechanical cardiac activity on arrival and underwent coronary angiography, with percutaneous coronary intervention where indicated, while circulation was maintained by an automated mechanical chest-compression device; this automated resuscitation device therefore represented the form of mechanical circulatory support used in this cohort.

The second cohort comprised 159 patients, who were grouped according to the location of cardiac arrest. The first group experienced out-of-hospital cardiac arrest, the second group experienced in-hospital cardiac arrest, and the third group experienced cardiac arrest in the catheterization laboratory. The two cohorts were constructed to address two distinct clinical questions: the prognostic relevance of resuscitation status and the prognostic relevance of arrest location. They were analyzed separately rather than pooled; consequently, they partially overlap in patients, no individual comparison includes any patient more than once, and no estimates were combined across cohorts.

The difference in cohort sizes reflects the inclusion criteria: patients whose cardiac arrest occurred in the catheterization laboratory were included in the location-based analysis but were excluded from the analysis based on pre-procedural resuscitation status, as they were neither resuscitated prior to the procedure nor transported to the catheterization laboratory in ongoing cardiac arrest.

Across both analyses, the primary outcomes were in-hospital mortality and post-resuscitation neurological status. Secondary outcomes included left ventricular ejection fraction and place of death (catheterization laboratory or hospital department).

Additional variables analyzed included resuscitation duration (minutes from cardiac arrest to restoration of mechanical cardiac activity). In cases with multiple resuscitation episodes, the cumulative resuscitation time was calculated. Other analyzed variables included initial lactate levels, age, sex, and the culprit vessel responsible for the acute coronary syndrome.

1. Although validated post-arrest neurological scales (e.g., the Cerebral Performance Category, modified Rankin Scale, and Glasgow Outcome Scale) exist, they could not be reliably applied retrospectively because the prospectively documented functional-status data they require were not consistently available in the clinical records. Patients were therefore divided into three pragmatic groups according to post-resuscitation neurological status: Coma.
2. Mild to moderate neurological disability (disorientation, altered mental status, personality changes, restlessness).
3. No evident neurological sequelae after resuscitation.

Neurological outcome could be determined only in patients who survived and for whom post-resuscitation neurological status was documented in the hospital records; survivors with missing neurological documentation were not included in the neurological outcome analysis. Accordingly, neurological outcome percentages are expressed relative to the number of survivors with available neurological data rather than to all survivors.Statistical analyses were performed using IBM SPSS Statistics (version 23; IBM Corp., Armonk, NY, USA). Continuous variables are presented as mean ± standard deviation, and categorical variables as absolute numbers and percentages. The distribution of continuous variables was assessed for normality using the Shapiro–Wilk test. As continuous variables were non-normally distributed, they were compared using the Mann–Whitney U test between the two resuscitation-status groups (RES vs. ARD) and the Kruskal–Wallis test among the three arrest-location groups (OHCA, IHCA, and CLCA). Categorical variables (including in-hospital mortality, post-resuscitation neurological status, and culprit-vessel distribution) were compared using the Pearson chi-square test, with Fisher’s exact test applied when expected cell counts were less than five. A two-sided *p*-value < 0.05 was considered statistically significant. Missing data were not imputed, and all analyses were performed on available cases; left ventricular ejection fraction was available in 5 of the 22 ARD patients (echocardiography performed in the emergency department in this subgroup) and in all RES patients, and post-resuscitation neurological status was documented only in a subset of survivors. No formal correction for multiple comparisons was applied; given the exploratory nature of the analyses, the reported *p*-values are considered hypothesis-generating and were interpreted with caution. As this was a retrospective analysis of all consecutive eligible patients treated during the study period, no a priori sample-size or power calculation was performed, and the study may be underpowered to detect differences in some subgroup comparisons, particularly those involving the small ARD and CLCA groups.

## 3. Results

### 3.1. Demographics

The study population included 159 patients, of whom 107 were male and 52 were female. The mean age of the study population was 66.9 ± 12.7 years. The mean age among male patients was 64.3 ± 11.4 years, whereas female patients were older, with a mean age of 72.3 ± 13.6 years.

All patients were admitted during or after cardiopulmonary resuscitation and were diagnosed with acute coronary syndrome (ACS)—either ST-segment elevation myocardial infarction (STEMI) or high-risk non-ST-segment elevation myocardial infarction (NSTEMI)—based on pre-hospital electrocardiography performed by emergency medical services or ECG obtained upon arrival to the emergency department.

Among the 159 patients, 80 (50.3%) had STEMI and 79 (49.7%) had NSTEMI. On invasive coronary angiography, significant coronary artery disease (defined as a diameter stenosis of 50% or more, with left main disease counted as two-vessel disease) was three-vessel in 85 patients (53.5%), two-vessel in 39 (24.5%), and single-vessel in 27 (17.0%), whereas no significant obstructive lesion was identified in 8 patients (5.0%). The infarct-related (culprit) lesion involved the anterior territory in 62 patients (39.0%), the inferior territory in 46 (28.9%), the lateral territory in 22 (13.8%), the anterolateral territory in 21 (13.2%), and the posterior territory in 4 (2.5%), while no culprit lesion could be identified in 4 patients (2.5%). As the entire cohort comprised patients resuscitated from cardiac arrest, all were classified as Killip class IV on admission.

### 3.2. Survival in Refractory Cardiac Arrest

The first cohort included 131 patients. The RES group comprised 109 patients (69.7% male; mean age 66.9 ± 11.8 years), while the ARD group included 22 patients (59.1% male; mean age 63.3 ± 15.1 years).

In-hospital mortality was 39.4% among patients resuscitated prior to catheterization compared with 86.4% among patients with refractory cardiac arrest requiring an automatic resuscitation device during the procedure (*p* < 0.001).

Only three patients in the ARD group were discharged from the hospital, two of whom remained in a coma due to severe post-hypoxic encephalopathy. Among survivors in the RES group, 66.2% had favorable neurological outcomes, 13.8% were discharged in a coma, and 20.0% had mild-to-moderate neurological disability.

Among deceased patients in the ARD group, 83.3% died in the catheterization laboratory due to refractory cardiac arrest, representing 68.2% of the entire ARD cohort, whereas 16.7% died in the intensive care unit. In the RES group, only 23.8% of deaths occurred in the catheterization laboratory (representing 9.2% of the entire RES group), while the remaining 76.2% occurred in cardiology departments during hospitalization.

Mean left ventricular ejection fraction (LVEF) in the RES group was 38.7 ± 9.9% (range 10–55%, median 40%). In the ARD group, five patients underwent echocardiography in the emergency department prior to coronary angiography, with a mean LVEF of 36.4 ± 17.8% (range 20–62%).

The mean resuscitation duration was 21.9 ± 17.3 min in the RES group compared with 66.1 ± 19.5 min in the ARD group (*p* < 0.001).

Mean lactate levels upon admission were 7.7 ± 3.7 mmol/L in the RES group and 10.7 ± 3.2 mmol/L in the ARD group (*p* = 0.006).

RES and ARD group comparison is shown in Table 1.

### 3.3. Survival According to Resuscitation Location 

In the second cohort (*n* = 159), stratified by location of cardiac arrest, 83 patients (52.2%) experienced out-of-hospital cardiac arrest (72.3% male; mean age 65.1 ± 12.6 years), 48 patients (30.2%) experienced in-hospital cardiac arrest (60.4% male; mean age 68.4 ± 11.9 years), and 28 patients (17.6%) experienced cardiac arrest in the catheterization laboratory (64.3% male; mean age 69.4 ± 13.9 years). 

In-hospital mortality was 39.8% in the out-of-hospital cardiac arrest group compared with 60.4% in the in-hospital cardiac arrest group (*p* = 0.001). The catheterization laboratory cardiac arrest group had the highest mortality rate at 78.6% (*p* < 0.001).

Neurological outcomes among survivors varied across groups. Good neurological recovery was observed in 54.5% of out-of-hospital cardiac arrest survivors and 68.4% of in-hospital cardiac arrest survivors, whereas 21.2% and 10.5%, respectively, had mild-to-moderate neurological disability, and 24.2% and 21.1%, respectively, were discharged in a coma (*p* = 0.21). Notably, all surviving patients from the catheterization laboratory cardiac arrest group had no neurological disability.

Mean LVEF was 38.8 ± 8.9% in the out-of-hospital group, 38.0 ± 13.6% in the in-hospital group, and 36.5 ± 11.3% in the catheterization laboratory group (*p* = 0.801).

Mean resuscitation duration was 27.7 ± 20.6 min in the out-of-hospital group, 34.7 ± 30.4 min in the in-hospital group, and 39.9 ± 24.9 min in the catheterization laboratory group (*p* = 0.237).

Mean lactate levels were 7.8 ± 3.8 mmol/L, 8.7 ± 3.8 mmol/L, and 7.2 ± 4.1 mmol/L, respectively (*p* = 0.432).

The comparison between OHCA, IHCA and CLCA is depicted in Table 2.

### 3.4. Culprit Vessel 

Analysis of the culprit coronary vessel in ACS did not demonstrate any significant association with primary or secondary outcomes. Specifically, the distribution of the culprit vessel did not differ significantly with respect to sex, resuscitation location, mechanical cardiac activity status in the catheterization laboratory, neurological outcome, or in-hospital mortality. 

Among the analyzed patients, the most frequently identified culprit vessel was the left anterior descending artery, accounting for 40.3% (62 patients). The right coronary artery was the second most common culprit vessel (27.8%, 45 patients), followed by the left circumflex artery (16.2%, 25 patients) and the left main coronary artery (13.0%, 20 patients). The ramus intermedius and saphenous vein graft were identified as the culprit vessel in 0.6% of cases each (1 patient each).

## 4. Discussion

### 4.1. Resuscitation Status at Angiography: Clinical Implications and Prognostic Significance

Contemporary guidance only partially addresses the invasive management of cardiac arrest complicating ACS, as most recommendations focus on patients who have already achieved return of spontaneous circulation (ROSC), while evidence remains limited in those who do not achieve sustained ROSC. Current guidance generally supports immediate invasive coronary angiography (ICA), with PCI when indicated, in post-ROSC patients with a high probability of acute coronary occlusion, particularly in the presence of persistent ST-segment elevation or equivalent findings, hemodynamic or electrical instability [4,5,6]. By contrast, in resuscitated patients without ST-segment elevation, management is typically individualized according to the overall clinical picture, including hemodynamic and neurological status. In hemodynamically stable OHCA patients without ST-segment elevation, routine immediate ICA has not shown benefit over a delayed or selective strategy in randomized trials such as COACT and TOMAHAWK [7,8]. This absence of benefit has proven durable: in the recently reported 5-year follow-up of COACT, long-term survival remained comparable between the immediate and delayed strategies (54.8% vs. 51.8%; HR 0.95; 95% CI 0.74–1.23), reinforcing that a routine immediate invasive approach confers no long-term advantage in resuscitated patients without ST-segment elevation [9]. In such presentations, early evaluation should also consider non-coronary causes of arrest. Illustrating this more selective management pathway, Voiçu et al. reported that among resuscitated OHCA patients with a non-shockable initial rhythm and no ST-segment elevation, ACS was identified in only 1% of cases, with no survivors in that subgroup [5]. However, our cohort represents a less clearly defined subgroup in whom ACS is highly likely, but ROSC is not achieved prior to catheterization, leaving the role of invasive management uncertain. For these patients, the balance between potential benefit and overall futility remains poorly defined, and the available evidence is limited largely to case reports and case series.

In patients with refractory cardiac arrest and a high suspected probability of ACS, ICA during ongoing mechanical cardiopulmonary resuscitation (mCPR) may represent a salvage invasive strategy. Published experience suggests that this approach is technically feasible, with devices such as LUCAS allowing acquisition of standard angiographic views during ongoing continuous chest compressions [10]. mCPR provides maintenance of systemic circulation during interventional procedures, ensuring adequate systemic blood pressure in most patients and producing sufficient coronary perfusion, albeit with variable success [11,12,13]. Nevertheless, this strategy remains procedurally challenging, as it requires precise device positioning, may increase radiation exposure and the risk of compression-related injury, and potentially limit operator maneuverability and certain angiographic projections [10]. Beyond mechanical compression alone, extracorporeal cardiopulmonary resuscitation (ECPR) has emerged as a further escalation, providing more complete circulatory support and enabling coronary angiography to be performed under sustained perfusion in patients who do not achieve ROSC. In a recent single-center cohort of 92 ECPR patients, angiography revealed significant coronary artery disease in 94% and a culprit lesion in 78%, indicating a high burden of treatable coronary pathology in this population; early angiography was associated with lower in-hospital mortality, an effect concentrated in patients with ST-segment elevation, although survival to discharge remained only 18% [14]. These findings reinforce the rationale for invasive evaluation when ACS is strongly suspected, while underscoring the limited survival that persists even with maximal circulatory support. In contrast to this extracorporeal approach, the refractory-arrest patients in our cohort were supported by mechanical resuscitation alone; whether escalation to ECPR would have altered their outcomes cannot be determined from our data and warrants further study.

Despite its technical feasibility, whether this approach translates into meaningful clinical benefit remains far less certain. Our findings indicate that resuscitation status at arrival to the catheterization laboratory is not merely a procedural descriptor, but a marker of arrest severity. Patients requiring ongoing device-assisted CPR during coronary angiography had substantially higher in-hospital mortality and worse neurological outcomes than those who had achieved preprocedural ROSC. This interpretation is reinforced by the longer resuscitation times and higher admission lactate levels observed in the refractory-arrest group, both of which are consistent with a more prolonged low-flow state and more severe systemic and cerebral hypoperfusion. In this context, preprocedural ROSC appears to represent an important prognostic threshold in ACS patients undergoing invasive evaluation. The findings of this study are broadly consistent with prior data showing significantly lower survival when refractory OHCA persists to an earlier point in the clinical course, namely, hospital arrival, than when ROSC has already been achieved, although a favorable neurological outcome can still occur among survivors [15]. Our data also align with prospective evidence demonstrating that longer CPR duration is independently and inversely associated with both survival and neurologically favorable outcome after OHCA [16]. Collectively, preprocedural resuscitation status in ACS patients with cardiac arrest appears to represent an important prognostic marker of both mortality and neurological outcome, with persistence of cardiac arrest up to catheterization identifying a distinctly high-risk subgroup characterized by limited survival and poor neurological recovery.

### 4.2. Cardiac Arrest Location and Clinical Outcomes

The second major finding is the association between cardiac arrest location and in-hospital mortality. In our cohort, mortality rose stepwise from OHCA to IHCA and was highest when arrest occurred in the catheterization laboratory itself. In contrast, neurological outcomes among survivors did not deteriorate in parallel; notably, all surviving patients from the catheterization laboratory arrest group had favorable neurological outcomes, although this observation must be interpreted cautiously given the small sample size and the non-significant between-group comparison. These findings suggest that arrest location is not just a descriptive variable, but a marker of the clinical context and severity surrounding the arrest. Furthermore, the lack of significant differences in LVEF, resuscitation duration, and lactate levels between groups suggests that arrest location captures prognostically relevant factors not fully reflected by conventional measured variables.

The higher in-hospital mortality observed in IHCA, compared with OHCA, may partly reflect the older age of patients in our cohort and a greater comorbidity burden. Although comorbidity burden was not assessed in the present study, this interpretation is nevertheless consistent with previous research highlighting the complex clinical context of IHCA patients [17,18]. In addition, OHCA patients included in the present cohort had necessarily survived to hospital admission and invasive evaluation, introducing a degree of selection that may also have contributed to their comparatively lower mortality.

In-laboratory cardiac arrest may represent a distinct clinical entity rather than simply a subset of in-hospital cardiac arrest, as, despite occurring in a highly monitored procedural environment, it often arises in patients undergoing urgent or emergent catheterization for STEMI, cardiogenic shock, or other high-risk interventions, and therefore frequently reflects severe underlying hemodynamic instability and clinical acuity [19]. Outcomes in this setting are also notably heterogeneous. In a GWTG (Get With The Guidelines)–Resuscitation registry analysis of 4787 cardiac arrests occurring in cardiac catheterization laboratories across 231 hospitals, the median risk-adjusted survival to discharge was 36%, varying from 20% to 52% across hospital tertiles [20]. These findings support the view that catheterization-laboratory cardiac arrest should not be considered a uniform condition and that prognosis is likely shaped by the underlying clinical scenario, arrest characteristics, and the resuscitation environment, rather than by location alone. Additionally, in the present study, an apparent dissociation was found between neurological outcomes and mortality, which may suggest that determinants of survival and of neurological preservation are not identical. While catheterization-laboratory arrest may reflect extreme hemodynamic compromise and poor overall survival, immediate recognition and treatment in a closely monitored setting may help preserve neurological viability among survivors [21,22].

Clinically, these findings suggest that arrest location may contribute to early prognostic framing but should not be interpreted in isolation or used as a standalone marker of futility. Cardiac arrest location in ACS patients undergoing coronary angiography appears to function as a contextual prognostic marker, reflecting differences in underlying clinical acuity, selection, and resuscitation circumstances.

### 4.3. Limitations

This study is limited by its retrospective, single-center design, small subgroup sizes, particularly in the ARD and catheterization-laboratory arrest groups, and the absence of multivariable adjustment, leaving the possibility of residual confounding. Because all comparisons are univariable and the groups differed substantially in arrest duration, admission lactate, and overall clinical acuity, the findings should be interpreted as associations rather than independent prognostic effects; the small subgroup sizes further preclude reliable multivariable or propensity-based modeling. The two cohorts were analyzed separately to address distinct clinical questions and partially overlap in patients; they are therefore not statistically independent and were not pooled and should not be interpreted as such. In addition, comorbidity burden was not systematically assessed, neurological outcome was classified using a pragmatic non-validated scheme, and the OHCA cohort was subject to survival selection before hospital admission and invasive evaluation. Moreover, several variables of prognostic relevance were not available in the dataset, including specific cardiovascular risk factors (hypertension, diabetes mellitus, dyslipidaemia, and prior coronary artery disease), the initial cardiac-arrest rhythm (shockable versus non-shockable), and the time from cardiac arrest to coronary angiography; these omissions limit interpretation of the reported outcomes and should be addressed in future prospective studies. Because validated neurological instruments could not be reliably applied retrospectively, the neurological findings are exploratory, limited in reproducibility, and not directly comparable with studies using standardized tools such as the CPC, mRS, or GOS.

## 5. Conclusions

Among patients with acute coronary syndrome presenting with cardiac arrest, both resuscitation status before catheterization and the location of cardiac arrest were important markers associated with clinical outcomes. Patients requiring ongoing mechanical resuscitation during coronary angiography experienced markedly higher mortality, whereas patients resuscitated prior to catheterization had significantly better survival and neurological recovery. Cardiac arrest occurring within the catheterization laboratory was associated with the highest mortality, although surviving patients demonstrated favorable neurological outcomes. These findings may assist clinicians in risk stratification and decision-making regarding invasive management in critically ill ACS patients.

## Figures and Tables

**Table 1:** Clinical characteristics and outcomes according to resuscitation status before catheterization.

Variable	RES Group (*n* = 109)	ARD Group (*n* = 22)	*p*-Value
Male sex, n (%) Age, years (mean ± SD) In-hospital mortality, n (%)	76 (69.7)	13 (59.1)	0.33
	66.9 ± 11.8	63.3 ± 15.1	
	43 (39.4)	19 (86.4)	<0.001
Death in catheterization laboratory, n (% of deaths) Death in ICU/department, n (% of deaths)	10 (23.8)	15 (83.3)	<0.001
	33 (76.2)	3 (16.7)	—
Favorable neurological outcome, n (%) Mild–moderate neurological deficit, n (%) Discharged in coma, n (%)	44 (66.2)	1 (33.3)	0.086
	13 (20.0)	0	—
	9 (13.8)	2 (66.7)	—
LVEF%, mean ± SD Resuscitation time, min (mean ± SD)	38.7 ± 9.9	36.4 ± 17.8 †	
	21.9 ± 17.3	66.1 ± 19.5	<0.001
Lactate at admission, mmol/L (mean ± SD)	7.7 ± 3.7	10.7 ± 3.2	0.006

Note: In-hospital mortality is calculated relative to the whole group; death location relative to deceased patients; and neurological outcome relative to survivors (RES, *n* = 66; ARD, *n* = 3), as neurological status was assessed only in survivors. † LVEF available in 5 of 22 ARD patients.

**Table 2:** Clinical outcomes according to location of cardiac arrest.

Variable	Out-of-Hospital (*n* = 83)	In-Hospital (*n* = 48)	Cath Lab (*n* = 28)	*p*-Value
Male sex, n (%) Age, years (mean ± SD) In-hospital mortality, n (%)	60 (72.3)	29 (60.4)	18 (64.3)	0.35
	65.1 ± 12.6	68.4 ± 11.9	69.4 ± 13.9	
	33 (39.8)	29 (60.4)	22 (78.6)	<0.001
Favorable neurological outcome, n (%) Mild–moderate neurological deficit, n (%)	18 (54.5)	13 (68.4)	6 (100)	0.21
	7 (21.2)	2 (10.5)	0	—
Discharged in coma, n (%) LVEF%, mean ± SD Resuscitation time, min (mean ± SD)	8 (24.2)	4 (21.1)	0	—
	38.8 ± 8.9	38.0 ± 13.6	36.5 ± 11.3	0.801
	27.7 ± 20.6	34.7 ± 30.4	39.9 ± 24.9	0.237
Lactate at admission, mmol/L (mean ± SD)	7.8 ± 3.8	8.7 ± 3.8	7.2 ± 4.1	0.432

Note: Neurological outcome was assessed only in survivors and is expressed relative to survivors with documented post-resuscitation neurological status; survivors with missing neurological documentation were not included.

## Data Availability

All data is available in the research site PC, secured by password as required by the ethics committee, and is not shareable beyond this article.

## Abbreviations

The following abbreviations are used in this manuscript:

ACS	Acute coronary syndrome
PCI	Percutaneous coronary intervention
RES	Previously resuscitated patients currently with mechanical heart activity
ARD	Patients undergoing resuscitation in the catheterization laboratory with automatic resuscitation device
LVEF	Left ventricular ejection fraction
OHCA	Out-of-hospital cardiac arrest
IHCA	In-hospital cardiac arrest
CLCA	Cardiac arrest in catheterization laboratory
ROSC	Return of spontaneous circulation
